# REACTANCE/HEIGHT AS A POTENTIAL BIOIMPEDANCE INDICATOR OF FRAILTY AMONG COMMUNITY-DWELLING OLDER ADULTS

**DOI:** 10.1093/geroni/igae098.4061

**Published:** 2024-12-31

**Authors:** Kworweinski Lafontant, Estefania Zamarripa, Abigail Tice, Amber Blount, Jethro Raphael Suarez, Dahee Kim, Jeffrey Stout, Ladda Thiamwong

**Affiliations:** University of Central Florida, Orlando, Florida, United States; University of Central Florida, Orlando, Florida, United States; University of Central Florida, Orlando, Florida, United States; University of Central Florida, Orlando, Florida, United States; University of Central Florida; University of Central Florida, Orlando, Florida, United States; University of Central Florida, Orlando, Florida, United States; University of Central Florida, Orlando, Florida, United States

## Abstract

Frailty is prevalent among older adults and is characterized by reductions in physical function and muscle quality. Despite the emerging clinical utility of bioelectrical impedance analysis (BIA), little is known about how bioimpedance indices, such as phase angle (PhA), Levi’s Muscle Index (LMI), reactance/height (Xc/Height), and resistance/height (R/Height), relate to physical function (i.e., handgrip strength, postural sway, Timed-Up-and-Go, Sit-to-Stand, Short Physical Performance Battery) and frailty. This study aimed to investigate these relationships in community-dwelling older adults. We cross-sectionally compared physical function measures and bioimpedance indices between frail, pre-frail, and robust categories as determined by the FRAIL questionnaire in 150 community-dwelling older adults (female, n = 137; age = 73.9 ± 6.9 years; BMI = 30.5 ± 6.4 kg/m2) using Kruskal-Wallis H and one-way ANOVA tests. Spearman rho (ρ) and Pearson r correlation coefficients were used to assess relationships between variables. PhA (r = -0.15, p = 0.07) and LMI (ρ = 0.05, p = 0.57) were not significantly associated with FRAIL scores, unlike Xc/Height (r = -0.29, p < 0.001) and R/Height (r = -0.20, p = 0.02). Among the bioimpedance indices, only Xc/Height differed significantly between frailty categories (F(2,147) = 6.55, p = 0.002) while all physical function assessments except postural sway differed between the categories (p < 0.05). These results suggest that only Xc/Height may be indicative of frailty among older adults. However, these conclusions may be limited by the use of the FRAIL questionnaire; future research should compare LMI and PhA using multiple frailty assessments.

